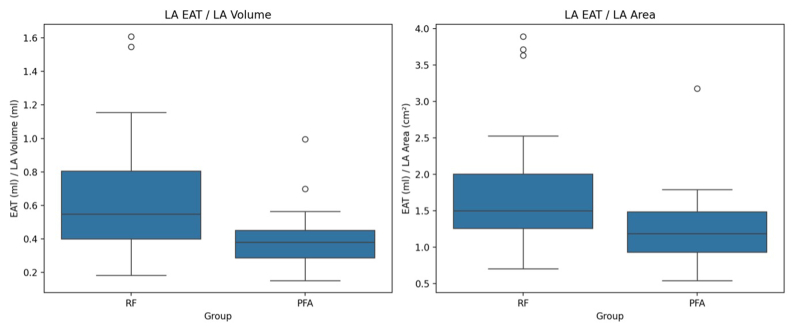# Epicardial adipose tissue and ablation outcomes in obese patients with paroxysmal atrial fibrillation: A comparison of pulsed field and radiofrequency ablation

**DOI:** 10.1016/j.hroo.2025.09.020

**Published:** 2025-09-25

**Authors:** Florian Englert, Theresa Obermeyer, Fabian Bahlke, Miruna Popa, Hannah Krafft, Alex Tunsch Martinez, Jan Syväri, Madeleine Tydecks, Dominic Dischel, Eva Koops, Theresa Reiter, Marta Telishevska, Sarah Lengauer, Kenno Bressem, Martin Hadamitzky, Gabriele Hessling, Isabel Deisenhofer, Nico Erhard

**Affiliations:** 1 Department of Electrophysiology, German Heart Center Munich, TUM University Hospital, Technical University of Munich, Munich, Germany; 2 Institute for Cardiovascular Radiology and Nuclear Medicine, German Heart Center Munich, TUM University Hospital, Technical University of Munich, Munich, Germany

**Keywords:** Pulsed field ablation, Radiofrequency, Epicardial adipose tissue, Atrium, Body mass index, Photon-counting computed tomography, Atrial fibrillation

## Abstract

**Background:**

Preclinical studies showed inconsistent results regarding the influence of adipose tissue on effective pulsed field ablation (PFA), raising questions about its efficacy in patients with elevated epicardial adipose tissue (EAT) levels.

**Objective:**

Elevated EAT levels may lead to higher atrial fibrillation (AF) recurrence rates after pulmonary vein isolation using PFA than high-power, short-duration radiofrequency (RF) ablation.

**Methods:**

103 patients with body mass index of >29 kg/m^2^ with paroxysmal or short-term persistent AF who underwent first-time AF ablation were prospectively enrolled (PFA n = 41; RF n = 62). All patients received preablation photon-counting computed tomography imaging to volumetrically quantify left and right atrial EAT levels. PFA was performed using a pentaspline catheter, and RF ablation was performed using high-power, short-duration energy.

**Results:**

Median EAT volumes were 71.85 mL (interquartile range 50.35–93.35 mL) in the RF group and 65.61 mL (interquartile range 40.45–90.8 mL) in the PFA group (*P* = .1352). Median follow-up was 367 days, excluding a 6-week blanking period. Atrial arrhythmia recurrence at 1 year was 33.87% in the RF group vs 17.07% in the PFA group (*P* = .077). Cox regression showed that, in the PFA group, left atrial EAT was the only significant predictor of recurrence (hazard ratio 1.06; 95% confidence interval 1.01–1.12; *P* = .022), corresponding to a 6.2% increased risk per mL. In the RF group, left atrial EAT was not significantly associated with recurrence (hazard ratio 1.00; 95% confidence interval 0.97–1.03; *P* = .846).

**Conclusion:**

PFA showed good 1-year results after pulmonary vein isolation in patients with a body mass index of >29 kg/m^2^. However, EAT may have a more significant impact on AF recurrences after PFA than RF ablation.

**Trial Registration Number:**

NCT06559787

## Graphical abstract

**Figure:**
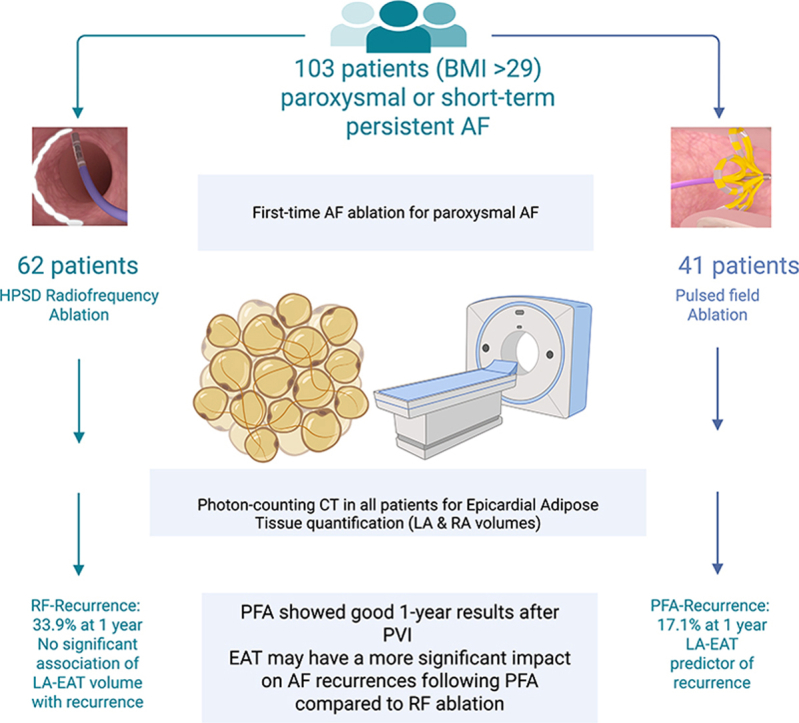

**Key Findings**

▪ Photon-counting computed tomography enabled precise volumetric quantification of left and right atrial epicardial adipose tissue (EAT) in all patients.
▪ Recurrence rates were lower after pulsed field ablation (PFA) than high-power, short-duration radiofrequency (RF) (17.1% vs 33.9% at 1 year).
▪ Left atrial EAT volume was an independent predictor of arrhythmia recurrence after PFA (hazard ratio 1.06; 95% confidence interval 1.01–1.12; *P* = .022).
▪ In the RF group, EAT volume showed no significant association with recurrence.

## Introduction

Left atrial (LA) catheter ablation has become well established to treat atrial fibrillation (AF), and technological advancements have made AF ablation safe and effective.^1^^,^^2^ Overcoming the limitations of pulmonary vein isolation (PVI) and achieving consistent ablation outcomes are still challenging, and individual atrial substrate characteristics such as scarring, wall thickness, LA size, and epicardial adipose tissue (EAT) play important roles when determining AF ablation success.^3^, ^4^, ^5^, ^6^, ^7^

High body mass index (BMI) levels have been associated with an increased risk of AF recurrence after catheter ablation where EAT likely plays an important contributing role.^8^, ^9^, ^10^ Most studies suggest a potential proarrhythmogenic effect of EAT through different inflammatory pathways that also influence success after catheter ablation by infiltration of the myocardium, causing less durable lesion formation.^11^, ^12^, ^13^ The effect of pulsed field ablation (PFA) on fat tissue and its impact on lesion formation has only been investigated in a few preclinical studies with inconsistent results regarding the influence of adipose tissue on effective PFA energy delivery. These findings raise questions about its efficacy in patients with elevated EAT levels.^14^^,^^15^

We hypothesized that the influence of EAT on AF ablation may depend on the underlying energy source and investigated the relationship between EAT levels and ablation outcomes comparing PFA with high-power, short-duration (HPSD) radiofrequency (RF) ablation.

## Methods

### Patient population

Between 2022 and 2024, a total of 103 patients were prospectively enrolled at the German Heart Centre in Munich for this study. Patients were aged 18 years or older with a BMI of >29 kg/m^2^ and scheduled to undergo their first catheter ablation for paroxysmal AF or persistent AF of <2 months. Patients with long-standing persistent AF, previous LA catheter ablation, history of cardiac surgery, congenital heart defects, or contraindications to computed tomography (CT) imaging were excluded.

A written informed consent was obtained from all participants before enrollment. All patients underwent photon-counting CT (PCCT) to exclude LA thrombus formation and to generate high-resolution biatrial 3-dimensional (3D) reconstructions, including detailed visualization of EAT.

This study was approved by the local ethics committee (approval number 2022-243_1-S-NP, Ethics Committee of the Technical University of Munich) and was registered as part of the prospective AFAT study at ClinicalTrials.gov (NCT06559787). The study was conducted in strict accordance with the Declaration of Helsinki and Consolidated Standards of Reporting Trials guidelines.

### PCCT

All patients underwent atrial PCCT imaging 24 hours or less before AF ablation using the Siemens NAEOTOM Alpha CT scanner (Siemens Healthineers). The primary purposes were to exclude LA thrombus formation and to enable reconstruction of the LA and surrounding anatomic structures. The imaging protocol used a split-bolus contrast administration technique delivered through a cubital intravenous line.

The contrast protocol consisted of 3 phases: an initial bolus of 50 mL iodine contrast agent (flow rate 4 mL/s), followed by a second bolus of 150 mL contrast agent mixed with mL 0.9% saline solution at a ratio of 30%:70% at 3 mL/s and a final 30 mL saline chaser at 3 mL/s. Scan timing was optimized using a test bolus technique. The protocol included 2 sequential scans: an arterial phase scan followed by a targeted LA appendage scan 60 seconds after injection.

Technical acquisition parameters included an electrocardiography-triggered scan start at 70%, with patients positioned supine and arms elevated. Tube voltage was set to 140 kV, and tube current was automatically optimized for each patient using CareDose 4D (Siemens Healthineers). The scan was performed with a detector collimation of 144 mm × 0.4 mm and a pitch of 3.2 and a rotation time of 0.25 seconds.

### Semiautomated 3D atrial segmentation

PCCT atrial reconstructions were obtained using inHEART medical, a computer-based system designed to create a digital reconstruction of atrial or ventricular anatomy and substrate including wall thickness, scarring, and EAT. Epicardial fat was segmented as voxels with attenuation between −1000 and −10 Hounsfield units (HU) located between the atria (LA and right atrium [RA]) and the pericardium, after subtraction of the esophagus and the lungs.

To ensure comprehensive and consistent EAT quantification, we deliberately chose the pulmonary artery trunk as the superior anatomic boundary, given that it provides a clear, reproducible landmark on CT imaging.^16^

For the EAT assessment, the pericardium was automatically segmented using an artificial intelligence–driven algorithm developed by inHEART, covering the region from the pulmonary artery bifurcation down to the level of the mitral and tricuspid valves. The outputs were maps of epicardial fat thickness onto the LA and RA endocardium geometries, expressed in mm and converted into mL.

**Supplemental fig 1:**
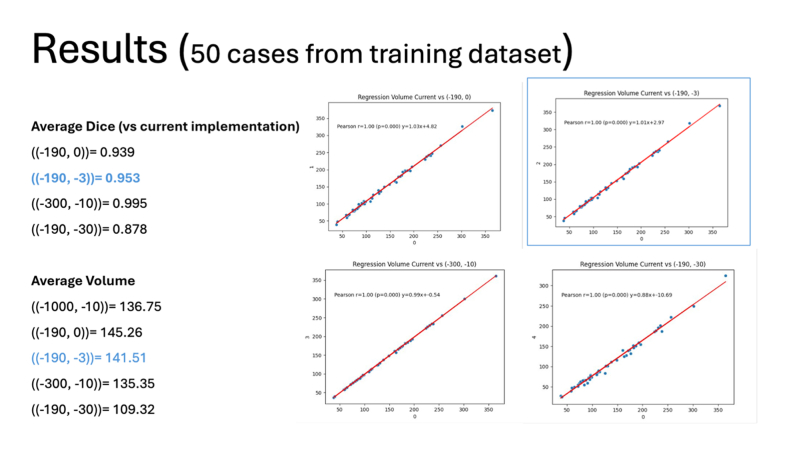


In our study, inHEART used a threshold of −1000 to −10 HU for epicardial fat segmentation to capture a wider spectrum of adipose tissue density, as enabled by the higher resolution and contrast differentiation of PCCT. To assess the validity of this wider range, inHEART performed a quantitative comparison using 50 annotated cases from a training dataset, comparing various threshold ranges including both the conventional (−190, −30 HU) and our selected setting (−1000, −10 HU) (Supplemental Figure 1).

### Preprocedural management

All patients were maintained on continuous oral anticoagulation for at least 4 weeks before the procedure and a minimum of 3 months after ablation. Antiarrhythmic drugs were discontinued at least 1 month before the procedure to avoid pharmacologic masking of arrhythmia substrates and prevent interference with procedural endpoints. Optimal medical therapy and comprehensive weight loss management strategies, including targeted exercise regimens and, if applicable, diabetes management, were encouraged during the preclinical visits.

All baseline demographic and clinical variables were obtained on the day of or the day before the first ablation procedure.

### Ablation procedure

Procedures were performed under monitored conscious sedation with a combination of midazolam, propofol, and fentanyl, ensuring patient comfort while maintaining adequate hemodynamic stability. Ultrasound-guided femoral venous access was established, with the insertion of 3 8F venous sheaths for RF procedures and 2 8F sheaths for PFA. Activated clotting time was maintained >300 seconds. All patients underwent PVI without additional lesions.

High-density 3D electroanatomic mapping of the LA was performed using either the EnSite X, HD Grid Mapping catheter (Abbott), or the CARTO 3, Pentaray mapping catheter (Biosense Webster). Wide antral circumferential point-by-point RF PVI was achieved using an irrigated-tip contact force–sensing ablation catheter. This was done using the HPSD protocol, as previously published by our group, with minor adaptations after the introduction of new ablation catheters.^17^^,^^18^ The used catheter, equipped with a pressure-sensitive tip, was used with the following standardized settings: 65 W power, a maximum temperature cutoff of 45°C, impedance cutoff at 200 Ω, and a maximum energy delivery duration of 15 seconds per application. The temperature cutoff function was enabled, whereas the TempGuard feature remained disabled. Irrigation was maintained at 18 mL/min during ablation, with pre- and postablation flushing durations of 1 second each. Lesions were delivered in a point-by-point manner using 65 W for 5–6 seconds on the posterior wall and 10–12 seconds on all other sites. AutoMark tags were configured with a diameter of 6 mm and were applied with a slight overlap to ensure lesion continuity.

PFA was performed using the FARAPULSE endocardial ablation system (Boston Scientific), which includes the FARAWAVE catheter, FARASTAR generator, and FARADRIVE sheath. The 12F over-the-wire catheter features 5 splines with 4 electrodes each, deployable in basket or flower configurations for circumferential contact with the pulmonary vein (PV) antra. Energy (2000 V) was delivered at least 8 times per vein using both configurations (basket and flower) to ensure complete PVI, confirmed by entrance and/or exit block. In cases of typical flutter, cavotricuspid isthmus ablation with RF was performed. In the PFA group, procedures were performed without additional electroanatomic mapping.

After ablation, groin access sites were closed using a Z-suture technique in both the RF and PFA groups. Transthoracic echocardiography was performed immediately after the procedure and again the following morning to evaluate for pericardial effusion or other complications. In the RF ablation group, all patients received pantoprazole 40 mg twice daily for 4 weeks.

### Follow-up

Ablation success was defined as the absence of continuous AF or atrial tachycardia for more than 30 seconds without the use of antiarrhythmic medications (class I or III), assessed at 3-, 6-, and 12-month follow-up visits using 7-day Holter electrocardiography monitoring.

A blanking period of 6 weeks was used after ablation. Complications within the first 30 days after ablation were evaluated based on in-hospital monitoring and subsequent follow-up assessments at 1 month or during any unplanned clinical visits.

### Statistical methods

Continuous variables are presented as mean ± standard deviation, and comparisons between 2 independent groups were performed using Student *t* test or Mann–Whitney U test, as appropriate. For variables that did not follow a normal distribution, the median and interquartile range (IQR) were reported. Categorical and binary variables are reported as counts (n) and percentages (%) and were compared between groups using the χ^2^ test or Fisher’s exact test, as appropriate.

Time to first recurrence of AF/atrial tachycardia was analyzed using the Kaplan–Meier method, with differences between groups assessed by the log-rank test.

A stratified Cox proportional hazards regression analysis was conducted to compare the association of EAT with RF and PFA groups. The Cox model estimated the hazard ratio (HR) and its 95% confidence interval (CI), adjusting for potential confounders including age, sex, hemoglobin A1c, BMI, diabetes, LA volume, and left ventricular ejection fraction (EF) while stratifying by ablation modality to account for baseline differences between groups.

A 2-sided *P* value of <.05 was considered statistically significant. All statistical analyses were conducted using SPSS software version 28.0 (IBM Corp, 2021) and R (R Foundation for Statistical Computing).

## Results

### Baseline characteristics

PFA was performed in 41 patients, HPSD ablation in 62 patients, with most patients having paroxysmal AF. Short-term persistent AF was present in 4 patients (9.8%) of the PFA group and 8 patients (12.9%) of the RF group. Baseline characteristics are presented in Table 1.

**Table 1:** Baseline patient characteristics

Characteristics	PFA (n = 41)	RF (n = 62)	*P* value
Age (y)	61.22 ± 11.01	66.60 ± 8.98	.008
Female frequency	17 (41.5%)	21 (33.9%)	.567
BMI (kg/m^2^)	31.92 ± 3.28	32.33 ± 3.59	.561
Body surface area (m^2^)	2.07 ± 0.15	2.11 ± 0.14	.299
Ejection fraction (%)	57.61 ± 4.92	53.92 ± 8.78	.022
Left atrial volume (mL)	71.79 ± 41.27	88.01 ± 27.87	.092
Baseline creatinine (mg/dL)	0.88 ± 0.22	0.97 ± 0.20	.033
Troponin (before the procedure, ng/L)	10.20 ± 6.29	12.38 ± 8.21	.153
Troponin (after the procedure, ng/L)	1431.30 ± 705.42	1480.58 ± 923.58	.775
Total cholesterol (mg/dL)	185.35 ± 37.66	174.47 ± 46.42	.217
HDL cholesterol (mg/dL)	53.36 ± 14.18	52.42 ± 14.56	.750
LDL cholesterol (mg/dL)	115.30 ± 34.33	109.37 ± 44.06	.472
HbA1c (%)	5.79 ± 0.65	5.99 ± 0.66	.145
Radiation dose (cGy × cm^2^)	473.31 ± 510.72	234.23 ± 253.47	.002
Fluoroscopy time (min)	15.19 ± 7.94	8.85 ± 6.84	.000
Hypertension	28.0 (68.3%)	48.0 (77.4%)	.423
Diabetes	7.0 (17.1%)	14.0 (22.6%)	.668
Coronary artery disease	6.0 (14.6%)	16.0 (25.8%)	.268
Previous stroke or TIA	4.0 (9.8%)	6.0 (9.7%)	.167
Heart failure (yes = 1; no = 0)	3.0 (7.3%)	15.0 (24.2%)	.052
Statin use (yes = 1; no = 0)	15.0 (36.6%)	33.0 (53.2%)	.146
Beta blockers (yes = 1; no = 0)	30.0 (73.2%)	51.0 (82.3%)	.304
Amiodarone after ablation (yes = 1)	2.0 (4.9%)	1.0 (1.6%)	.664
Groin complications (yes = 1)	5.0 (12.2%)	1.0 (1.6%)	.070

Values are mean ± standard deviation or n (%). *P* values from *t* test or Fisher’s exact/χ^2^ test, as appropriate. All data are baseline unless otherwise noted (postprocedure troponin, radiation dose, fluoroscopy time, groin complications).

BMI = body mass index; HbA1c = hemoglobin A1c; HDL = high-density lipoprotein; LDL = low-density lipoprotein; PFA = pulsed field ablation; RF = radiofrequency; TIA = transient ischemic attack.

The mean age was higher in the RF group (66.60 ± 8.98 years) than the PFA group (61.22 ± 11.01 years; *P* = .008). EF was lower in the RF group (53.92 ± 8.78% vs 57.61 ± 4.92%; *P* = .022). No significant differences were observed in total cholesterol, high-density lipoprotein cholesterol, or low-density lipoprotein cholesterol levels. In addition, there were no statistically significant differences in the rates of diabetes, coronary artery disease, or previous strokes between the 2 studied groups. Gender distribution and medication use also did not differ significantly.

### Ablation outcome

**Figure 1:**
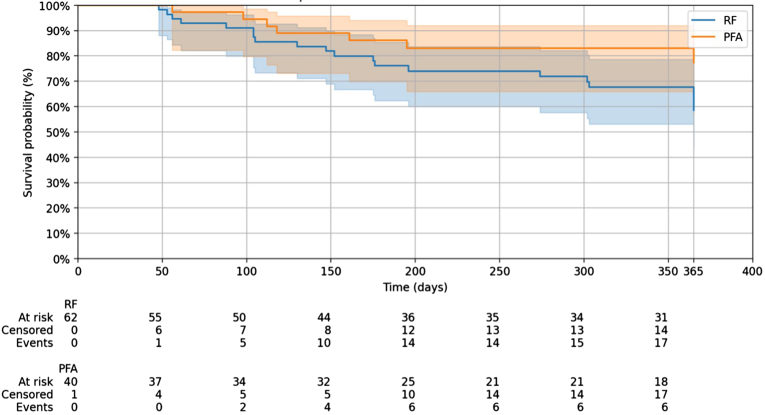
Kaplan–Meier curve of recurrence rates of PFA vs RF ablation groups. Patients treated with PFA *(orange)* demonstrated higher arrhythmia-free survival than those treated with high-power, short-duration RF ablation *(blue)* during 12 months of follow-up. Shaded areas represent 95% confidence intervals. Numbers at risk, censored patients, and events are displayed below the x-axis. PFA = pulsed field ablation; RF = radiofrequency.

Acute PVI was successfully achieved in all patients in both groups. The median follow-up period (excluding a 6-week blanking period) was 367 days (IQR 235–498) (PFA group 351 days [IQR 208–409]; RF group 378 days [IQR 364–494]). Overall recurrence rate at 1 year (excluding 6-week blanking period) was 27.18%. Recurrence of any atrial arrhythmia was observed in 33.87% of patients in the RF group and in 17.07% of patients in the PFA group; however, this difference did not reach statistical significance (log-rank test; *P* = .077) (Figure 1). Only 2 patients in the PFA group and 1 patient in the RF group (4.9% vs 1.6%; *P* = .664) received amiodarone after ablation owing to early recurrence. These patients were marked as recurrent in the survival analysis at the time of recurrence and after drug introduction.

In subgroup analyses, recurrence rates in the RF group did not differ significantly between patients with and without heart failure (*P* = .41). Among patients without heart failure, recurrence remained higher in the RF group than PFA (29.7% vs 15.6%; *P* = .04).

### EAT and BMI

**Figure 2:**
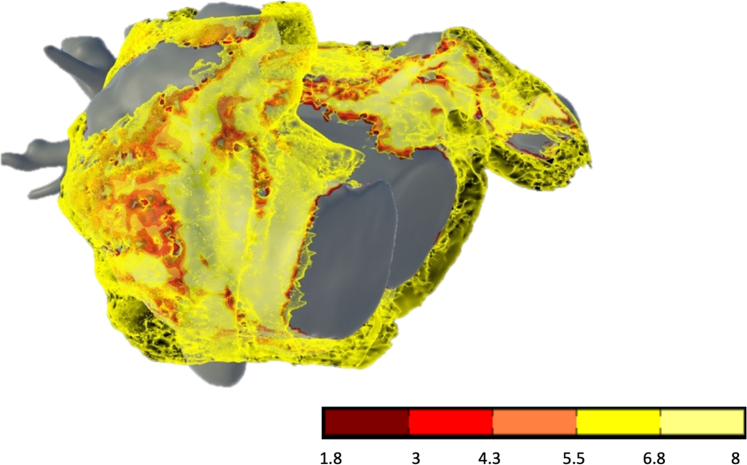
Biatrial epicardial adipose tissue (in *yellow*) quantification by photon-counting computed tomography and 3-dimensional segmentation (inHEART medical), shown in anteroposterior view. The underlying *red* and *yellow colors* represent atrial wall thickness (in mm), which was not used in this study. The depicted patient was from the pulsed field ablation group.

Volumetrically, EAT quantification was performed, and an explanatory representation of the 3D models created to precisely quantify RA and LA EAT levels in mL is shown in Figure 2. Visible atrial wall thickness was not used in the present analyses, but was displayed because it is automatically generated by the segmentation software and facilitates anatomic visualization.

**Figure 3:**
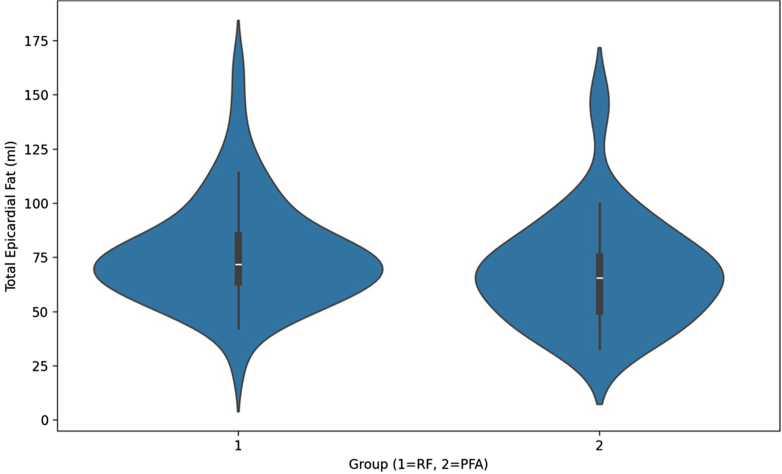
Distribution of total epicardial adipose tissue volume in patients undergoing first-time atrial fibrillation ablation, stratified by ablation modality. Group 1 represents high-power, short-duration radiofrequency ablation, and group 2 represents pulsed field ablation. The violin plots illustrate the distribution, median *(white dot)*, and interquartile ranges *(black bar)*. Median epicardial adipose tissue volumes did not differ significantly between groups.

**Figure 4:**
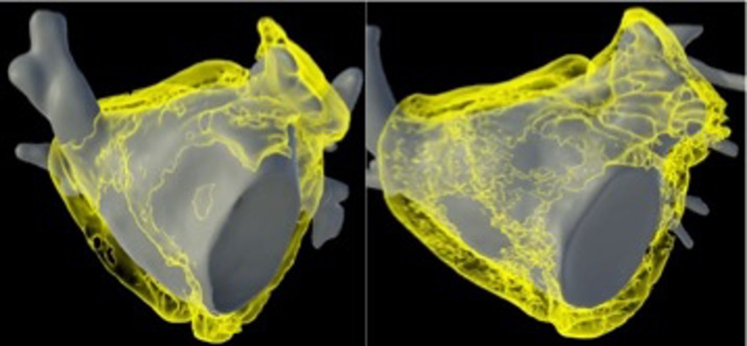
Left atrial volumetric quantification of atrial epicardial adipose tissue (29 mL left [pulsed field ablation group]; 51 mL right [radiofrequency group]) using photon-counting computed tomography imaging and 3-dimensional segmentation (courtesy of inHEART medical), shown in anteroposterior view. Epicardial adipose tissue is visualized in *yellow*.

The median total EAT volumes were 65.61 mL (IQR 40.45–90.8 mL) in the PFA group and 71.85 mL (IQR 50.35–93.35 mL) in the RF group (*P* = .1352) (Figures 3 and 4). The median LA EAT volume was 33.24 mL (IQR 23.62–40.82 mL) in the PFA group and 37.55 mL (IQR 30.47–43.22 mL) in the RF group (*P* = .112). The median RA EAT volume was 32.52 mL (IQR 25.53–38.51 mL) in the PFA group and 35.64 mL (IQR 31.16–43.29 mL) in the RF group (*P* = .084). The RF group demonstrated a significantly higher median EAT/LA volume (0.55 [IQR 0.40–0.81]) than the PFA group (0.38 [IQR 0.29–0.45]; *P* = .030). Similarly, the EAT-to-LA area ratio was greater in the RF group (1.50 [IQR 1.26–2.01]) than in the PFA group (1.19 [IQR 0.93–1.49]; *P* = .047). These findings are also presented in Table 2. The mean BMI was similar between groups, measuring 31.92 ± 3.28 kg/m^2^ in the PFA cohort and 32.33 ± 3.59 kg/m^2^ in the RF cohort (*P* = .561).

**Table 2:** RA EAT and LA EAT normalized to atrial size

LA and RA EAT	PFA (n = 41)	RF (n = 62)	*P* value
Median total EAT volumes (mL) (IQR)	65.61 (40.45–90.8)	71.85 (50.35–93.35)	.1352
Median LA EAT (mL) (IQR)	33.2 (23.6–40.8)	37.5 (30.5–43.2)	.112
Median RA EAT (mL) (IQR)	32.5 (25.5–38.5)	35.6 (31.2–43.3)	.084
LA EAT to LA volume ratio (mL/mL)	0.38 (0.29–0.45)	0.55 (0.40–0.81)	.03
LA EAT to LA area ratio (mL/cm^2^)	1.50 (1.26–2.01)	1.19 (0.93–1.49)	.047
LA EAT index (LA EAT volume/BSA) (mL/m^2^)	17.60 ± 6.28	18.50 ± 5.94	.526

Values are median (IQR) or mean ± standard deviation; *P* values from *t* test or Mann–Whitney *U* test. EAT volumes from photon-counting CT segmentation.

BSA = body surface area; EAT = epicardial adipose tissue; IQR = interquartile range; LA = left atrial; PFA = pulsed field ablation; RA = right atrial; RF = radiofrequency.

LA EAT index—calculated as LA EAT volume divided by body surface area—averaged at 18.2 mL/m^2^ overall. By ablation modality, the RF group showed a mean ± standard deviation of roughly 18.5 ± 5.9 mL/m^2^, whereas the PFA group had 17.6 ± 6.3 mL/m^2^. Statistical comparison indicated no difference between groups (*P* = .53).

**Figure 5:**
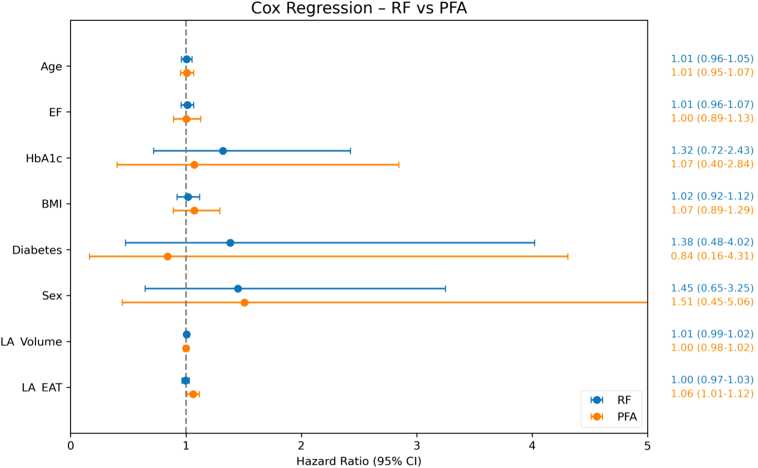
Forest plot displaying the influence of LA EAT and covariates on ablation outcomes after RF *(blue)* and PFA *(orange)* ablation. BMI = body mass index; CI = confidence interval; EAT = epicardial adipose tissue; EF = ejection fraction; HbA1c = hemoglobin A1c; LA = left atrial; PFA = pulsed field ablation; RF = radiofrequency.

Stratified Cox proportional hazards regression analysis, adjusted for potential confounders including age, sex, hemoglobin A1c, BMI, diabetes, LA volume, and left ventricular EF, revealed divergent associations between LA EAT volume and arrhythmia recurrence risk in the 2 cohorts (Supplemental Figure 2). In the PFA group, a significant association was found (HR 1.06; 95% CI 1.01–1.12; *P* = .022) (Figure 5) with each additional milliliter of EAT conferring a 6.2% increased risk of arrhythmia recurrence. In the RF group, increased LA EAT volume was not significantly associated with the risk of arrhythmia recurrence (HR 1.00; 95% CI 0.97–1.03; *P* = .846).

### Complications

Groin complications were observed in 5 patients (12.2%) of the PFA cohort and 1 patient (1.6%) of the RF cohort (*P* = .070). There was no major complication, including pericardial effusion or stroke, in either group.

## Discussion

To the best of our knowledge, this is the first prospective nonrandomized clinical study to investigate the effects of EAT on ablation outcomes after PVI in PFA compared with HPSD ablation and the first study to use PCCT imaging for systematic quantification of atrial EAT. Overall, PFA resulted in high rates of freedom from any atrial arrhythmia recurrences after 1 year in patients with a BMI of >29 kg/m^2^. However, our data suggest that atrial EAT levels may have a more significant influence on recurrence rates in PFA than HPSD RF ablation.

Recurrence of any atrial arrhythmia was observed more often in the RF group (33.87%) than in the PFA group (17.07%) without reaching statistical significance. Our results are in line with previous studies regarding recurrence rates after catheter ablation.^19^^,^^20^ It might be assumed that, among other factors that are discussed in more detail, baseline differences, including age, higher creatinine levels, and more advanced structural and functional heart disease in the RF group, could have contributed to the higher recurrence rates observed, even among patients without heart failure, irrespective of the energy source used.^21^, ^22^, ^23^

Our study was not powered to demonstrate the superiority of PFA over RF in patients with higher BMI levels, and more data from larger, randomized controlled trials or large registry studies are needed.

Previous studies have demonstrated that EAT can be a predictor of recurrence after catheter ablation for AF. Importantly, these investigations were largely based on patients undergoing RF or cryoballoon ablation, with no available data yet on PFA. A systematic review and meta-analysis showed that total EAT volume, LA EAT volume, and epicardial fat thickness were all associated with AF recurrence after ablation.^24^ Another recent meta-analysis highlighted that peri-LA EAT specifically seems to be a strong predictor of postablation recurrence.^25^^,^^26^ In these analyses, patients with persistent AF were also included, whereas, in the presented data, only patients with paroxysmal AF were included, which may explain the limited impact of EAT on recurrence observed in the RF group. Previous evidence suggests that, contrary to persistent AF, EAT parameters are associated less with AF recurrence after ablation in paroxysmal AF.^26^^,^^27^

To quantify EAT levels, PCCT represents an innovative imaging modality that offers enhanced spatial resolution and optimized spectral imaging, resulting in exceptional cardiac image quality, which precisely delineates the atrial substrate.^28^^,^^29^ PCCT also provides reduced image noise compared with conventional CT imaging, thereby enabling precise visualization of critical structures including the esophagus and EAT, while maintaining comparable radiation dosage.^30^ Moreover, advancements in segmentation software solutions enable the integration of semiautomated 3D reconstruction of cardiac anatomy into both research and clinical environments.^31^, ^32^, ^33^ This imaging modality is already well established for ventricular reconstructions in patients with ventricular arrhythmias, which allows detailed substation visualization including scar and wall thickness assessment. Previous studies have demonstrated benefits regarding outcome and procedural time when integrating ventricular reconstruction into the ablation workflow of ventricular tachycardias.^30^^,^^31^^,^^34^ To the best of our knowledge, no study has investigated the use of this imaging modality in the ablation setting of atrial arrhythmias.

To ensure comprehensive and consistent EAT quantification, we deliberately chose the pulmonary artery trunk as the superior anatomic boundary, given that it provides a clear, reproducible landmark on CT imaging. This area contains perivascular fat that may influence atrial electrophysiology and lesion formation, especially near the superior PVs. We aimed to create a standardized segmentation protocol to ensure the highest level of comparability between patients.

We hypothesized that EAT may influence ablation outcomes differently depending on the energy source used. Data from Gasperetti et al^15^ from an experimental preclinical model indicated a limitation of PFA in the presence of high amounts of fat within the ablation area if the fat layer was located between the catheter and the target tissue to be ablated, rather than intramyocardially. In contrast, other studies demonstrated improved lesion formation when intramyocardial fat influences the electrical field distribution and the resulting lesion shape.^14^^,^^35^ Ultimately, available data on this topic remain limited and inconsistent. In our study, Cox regression analysis revealed differing impacts of EAT on recurrence risk using the 2 ablation methods.

In our study, in the PFA group, LA EAT was the only significant predictor for recurrence: HR of 1.06 (95% CI 1.01–1.12; *P* = .022). In the RF group, increased LA EAT volume was not significantly associated with the risk of arrhythmia recurrence (HR 1.00; 95% CI 0.97–1.03; *P* = .846). These findings suggest that elevated EAT, particularly in the LA region, may interfere with PFA efficacy, potentially owing to its impact on the electric field distribution or insulation effects.^15^ RF ablation lesions seem to have fewer interactions with underlying fat tissue.^27^

Although conventional RF ablation is known to modulate atrial ganglionated plexi (GPs) through deeper thermal lesions,^36^ this effect was not evident in our results. A likely explanation is that we applied an HPSD RF protocol**,** which creates more superficial and less transmural lesions than conventional RF.^17^ As a result, autonomic modulation is reduced. In addition, the greater atrial EAT burden in the RF cohort may have offset any potential autonomic effects, thereby obscuring GP-related influences on recurrence outcomes. Endocardial PFA generally spares GPs with only minimal transient effects.^37^

In subgroup analyses, recurrence rates in the RF group did not differ significantly between patients with and without heart failure (*P* = .41), consistent with previous reports.^38^ Importantly, among patients without heart failure, recurrence rates remained higher in the RF group than the PFA group (29.7% vs 15.6%; *P* = .04).

Groin complications were more frequent in the PFA group than in the RF group, although no major complications occurred, likely owing to a small cohort. Large studies have not shown increased vascular risks with PFA despite BMI-related access complications.^39^^,^^40^

This study included patients with a BMI of >29 kg/m^2^, given that they have higher EAT levels, providing a strong data foundation. Weight loss strategies before and after the procedure remain crucial for optimal outcomes in patients with adiposity and AF.^41^^,^^42^

The findings of this study highlight the importance of LA EAT as a potential influence on AF recurrences after PFA, given its proximity to critical ablation targets in the PV antra. Strategies to mitigate the impact of EAT, such as refining catheter configurations or adjusting energy delivery parameters, may improve PFA efficacy even further in patients with a high BMI and higher EAT volumes.

### Limitations

The nonrandomized design and relatively small sample size introduce potential selection bias and limit generalizability. Although EAT was quantified using PCCT and 3D segmentation, other structural factors were not included, such as wall thinning or scar tissue. Differences in energy delivery methods (PFA vs RF) might influence outcomes beyond the impact of EAT alone. The study does not assess lesion durability, PV reconnection rates, or procedural success beyond recurrence rates. Arrhythmia recurrence detection may be incomplete owing to the lack of continuous monitoring. No systematic remapping data were obtained, and therefore, lesion extension or voltage reduction in relation to EAT could not be assessed. Statistical significance of the study’s main finding was borderline and should be interpreted cautiously. Although segmentation was performed by a third party and overseen by experienced radiologists, we were unable to assess interobserver variability in this study. We acknowledge that, owing to the limited sample size, our findings can and should only be considered hypothesis generating. We described a novel association in our study and aim for additional investigations in larger cohorts in the future.

## Conclusion

The findings of this study show that EAT has a more significant impact on arrhythmia recurrences using PFA than RF ablation. These findings warrant further investigation of the mechanisms of EAT affecting PFA energy delivery to develop strategies to overcome these challenges. Understanding the interactions between EAT and different ablation modalities could guide personalized treatment approaches for patients with AF.

## Disclosures

The authors have no conflicts of interest to disclose.

## Figures and Tables

**Supplemental fig 2:**