# Supplementary material for: Epicardial adipose tissue and ablation outcomes in obese patients with paroxysmal atrial fibrillation: A comparison of pulsed field and radiofrequency ablation
Source: Heart Rhythm O2. 2025 Sep 25;6(12):1901–10. doi: 10.1016/j.hroo.2025.09.020 (PMC12800804; doi:10.1016/j.hroo.2025.09.020)
Supplement: Supplemental material [file mmc1.docx]

Supplement 1: Quantitative comparison using 50 annotated cases from a inHEART© training dataset, comparing various threshold ranges including both the conventional (–190, –30 HU) and our selected (–1000, –10 HU) setting.

Supplement 2: Stratified Cox proportional hazards regression analysis, adjusted for potential confounders including age, sex, HbA1c, BMI, diabetes, LA volume and LVEF revealed divergent associations between LA EAT volume and arrhythmia recurrence risk in the two cohorts.
